# Contact Lenses as Ophthalmic Drug Delivery Systems: A Review

**DOI:** 10.3390/polym13071102

**Published:** 2021-03-30

**Authors:** Paola Franco, Iolanda De Marco

**Affiliations:** 1 Department of Industrial Engineering, University of Salerno, Via Giovanni Paolo II, 132, 84084 Fisciano (SA), Italy; pfranco@unisa.it; 2 Research Centre for Biomaterials BIONAM, University of Salerno, Via Giovanni Paolo II, 132, 84084 Fisciano (SA), Italy

**Keywords:** contact lenses, ophthalmic drug, polymeric support, ocular drug delivery

## Abstract

Ophthalmic drugs used for the treatment of various ocular diseases are commonly administered by eye drops. However, due to anatomical and physiological factors, there is a low bioavailability of the active principle. In order to increase the drug residence time on the cornea to adequate levels, therapeutic contact lenses have recently been proposed. The polymeric support that constitutes the contact lens is loaded with the drug; in this way, there is a direct and effective pharmacological action on the target organ, promoting a prolonged release of the active principle. The incorporation of ophthalmic drugs into contact lenses can be performed by different techniques; nowadays, the soaking method is mainly employed. To improve the therapeutic performance of drug-loaded contact lenses, innovative methods have recently been proposed, including the impregnation with supercritical carbon dioxide. This updated review of therapeutic contact lenses production and application provides useful information on the most effective preparation methodologies, recent achievements and future perspectives.

## 1. Introduction

The human eye is an extremely delicate organ, often prone to irritation, dryness and various diseases, such as glaucoma, cataracts, keratoconus, age-related macular degeneration, and many others. These ocular clinical conditions also affect patients’ quality of life. According to the World Health Organization, every five seconds a person in the world becomes blind; in addition, about 1.3 billion people suffer from vision impairments [1].

Nowadays, eye drops are the most widely used ocular drug delivery system; indeed, it is estimated that about 90% of ophthalmic drugs are administered in the form of eye drops [2,3,4]. Although this route of administration is well-accepted by patients, the ocular bioavailability of drugs administered with topical eye drops is very low, the numerous anatomical constraints, such as the corneal epithelium, blood–aqueous and blood–retinal barriers, hinder the correct and deep ocular permeation of the drug [5]. Also, considering the physiological factors, such as nasolacrimal drainage and blinking, a maximum of 5% of the drug dose contained in the ophthalmic drops reaches the deeper ocular tissues, while the residual dosage is lost due to tear drainage and absorption through the eye’s conjunctiva [6]. Consequently, the residence time of the necessary drug concentration on the cornea is inefficient, resulting in severe side effects. In order to maintain adequate therapeutic levels for a longer period of time, innovative ophthalmic drug delivery systems have recently been proposed to overcome the limitations associated with conventional formulations. To date, the most promising tool is the incorporation of active principles into contact lenses [7,8,9]. Although the primary use of contact lenses is related to the correction of ametropia, there is a growing interest in their application as therapeutic devices for several purposes: Maintaining corneal epithelial hydration, relieving eye pain, promoting corneal healing, as well as controlled drug administration for the treatment of ocular diseases [7,8,9,10].

**Figure 1:**
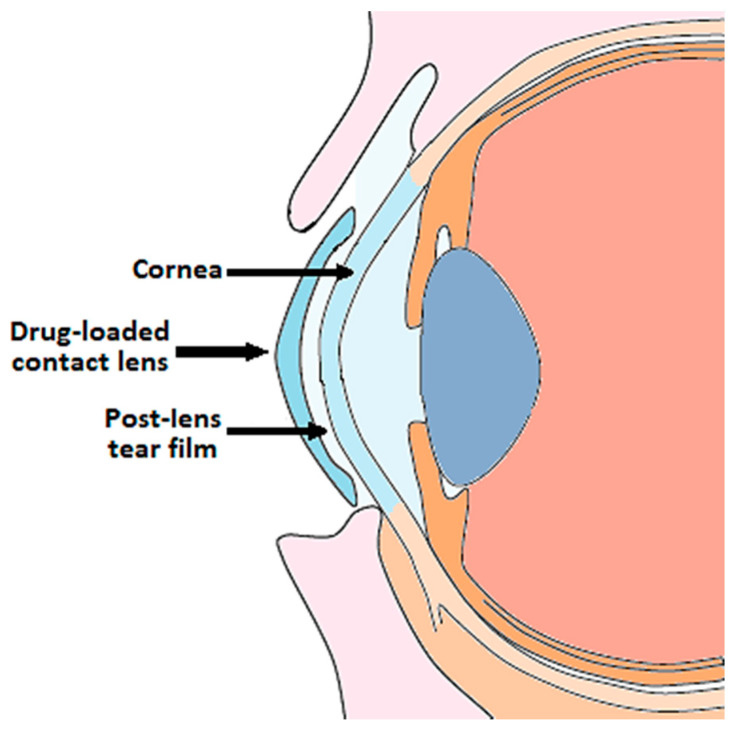
Ophthalmic drug delivery from contact lenses.

The incorporation of the drug into the lens matrix favors a prolonged release of the active principle towards the post-lens tear film in contact with the cornea, where the drug has to penetrate (Figure 1).

Achieving sustained or prolonged release of the ophthalmic drug from contact lenses allows to reduce the frequency of administration and the dose required to reach the desired therapeutic effect [5,8,11]. In addition, the lower drug loss in the case of therapeutic lenses, compared to the use of eye drops, leads to an increase in ocular bioavailability, which is still a challenge.

The use of contact lenses for therapeutic purposes is also very attractive because it is estimated that around 100 million people currently wear them, a number that will increase exponentially in the near future [9]. However, there are still some issues to be solved mainly related to the preparation and storage of drug-loaded lenses, or the non-use of contact lenses by the elderly population, more affected by ocular pathologies. Furthermore, to the best of our knowledge, no therapeutic contact lenses have been yet marketed, being at most in the preclinical or clinical study stage [12]. Nevertheless, it is clear that the benefits associated with the use of these new ocular formulas are relevant for the scientific advancement of both the polymeric and pharmaceutical fields. Therefore, in this review, the focus is on preparing therapeutic contact lenses using different techniques. The most advantageous or innovative methodologies are highlighted, as well as the different supports for loading the drug. This review aims to be a useful tool for future developments in the delivery of ophthalmic drugs for the treatment of ocular diseases.

## 2. Different Supports to Produce Therapeutic Contact Lenses

To date, most of the proposed ophthalmic drug delivery systems are polymer-based formulations [13,14]. In this context, the use of a wide variety of polymers has been explored, including natural, semisynthetic and synthetic ones [14]. While ensuring good biocompatibility, natural hydrophilic polymers such as alginate, and similarly, semisynthetic hydrophilic polymers, such as chitosan or gelatin, guarantee a good incorporation of water-soluble compounds, but are not able to favor a prolonged release of ophthalmic drugs [14]. Conversely, hydrophobic synthetic polymers, such as polycaprolactone (PCL), Eudragit or poly(lactide) (PLA)-based polymers, enable the modulation of drug release kinetics and reduce the initial burst effect due to the dissolution of the drug, loaded on the external surface, and not incorporated in the polymeric matrix [14]. However, a low loading efficiency of water-soluble drugs is generally obtained when these hydrophobic polymers are used as carriers. For this reason, hybrid polymeric carriers have also been proposed for the ophthalmic drug delivery, combining polymers of different nature, thus, improving the performance of the ocular drug delivery system [14].

Both reservoir and matrix systems have been applied as ocular therapeutic forms; substantially, in the first case, there is a core consisted of the active principle surrounded by a polymeric layer. While, in the other type, the drug is homogeneously dispersed in a polymeric matrix [13].

In Table 1, a list of the main polymers that usually constitute the network of the therapeutic contact lenses, in addition to other components present to a lesser extent is reported. Some details about physicochemical properties/characteristic features were also indicated for each polymer.

**Figure 2:**
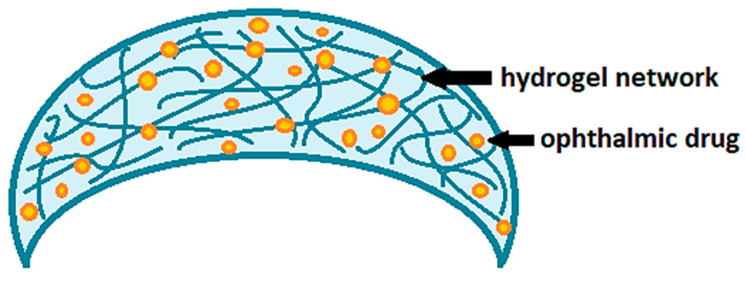
Drug-loaded soft contact lenses based on hydrogels.

Contact lenses loaded with drugs are certainly among the most innovative delivery systems proposed to improve corneal permeation and the bioavailability of ophthalmic drugs. Nowadays, conventional hydrogel-based soft contact lenses are the most proposed ones for therapeutic purposes [15,16,17], as also widely demonstrated by the studies reported in Table 1. Hydrogels are generally defined as polymer networks extensively swollen with water [18]. Due to the high porosity and surface area, hydrogels have the ability to incorporate active principles within their own network (Figure 2). Once the therapeutic hydrogel contact lenses are worn, the embedded drug is released to the post-lens tear fluid, thus, reaching the target tissue.

The hydrogels that are used to produce therapeutic soft contact lenses are generally synthetized by free radical polymerization [4,19,20,21,22,23] and ultraviolet light polymerization [22,24], as well as cast moulding [19,25,26]. The main network of hydrogels that constitutes soft contact lenses is usually based on poly(hydroxyethyl methacrylate) (HEMA) [22,27,28,29], especially methafilcon A [30,31,32]. Similarly, the marketing of hydrogel contact lenses based on silicone [24,28,33,34,35,36], also called polysiloxane, is also widespread today. Recently, hydrogels that respond to external stimuli, such as temperature and pH changes, have also been proposed for the delivery of ocular drugs [28,37]. For example, Kim et al. [28] prepared pH-sensitive hydrogels, which exhibited different swelling behaviors at different pH values in the range 5.8–8.0, and consequently, different drug release kinetics.

**Figure 3:**
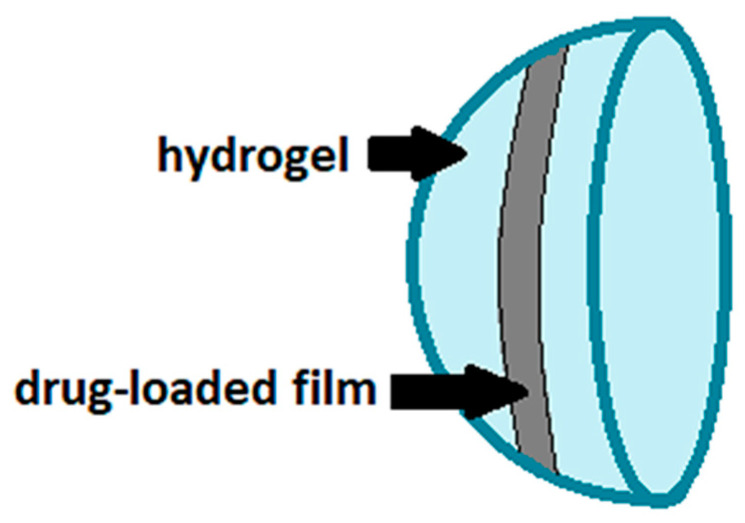
Drug-loaded film incorporated into hydrogel-based contact lenses.

Although the favorable properties of gels, such as biocompatibility, softness and flexibility, the penetration of the drug in soft contact lenses is influenced by multiple factors, including the water content in the polymeric network, the thickness of the lens and the molecular weight of the ophthalmic drugs to be incorporated [9]. Furthermore, any fast swelling of the hydrogel when in contact with an aqueous environment can cause a too rapid release of the ophthalmic drug, which is undesirable especially for the treatment of chronic diseases. Therefore, modifications of the polymeric network constituting the contact lens or the use of different polymeric supports are currently being studied [9]. In this scenario, the use of polymeric thin films is included, which have recently been proposed as therapeutic contact lenses after drug impregnation [38,39,40] or as a drug-loaded platform embedded in hydrogel-based lenses (Figure 3) [27,29,30,31,32]. In the latter case, ultraviolet light polymerization is generally employed to coat both sides of the drug-loaded film with the gelling polymers [27,29,30]. Alternatively, the solution containing the drug and the film-forming polymers can be pipetted directly onto the concavity of the hydrogel lens; after the solvent evaporation, the ultraviolet coating method is used to cover the side of the film not yet encapsulated in the lens [31,32].

Significant efforts have also been made over the years to improve the properties of contact lenses [40]. Since eye dryness is the most common discomfort that prompts consumers to cease wearing contact lenses, Yu et al. [40] suggested a novel approach to improve the wettability and lubrication of commercial lenses. The proposed method involved the polymerization of a thin film of hydrophilic poly-dimethylacrylamide (DMA) on the surface of the contact lens, which has been soaked in a liquid solution, containing azobisisobutyronitrile (AIBN) as a hydrophobic thermal initiator. This approach is effective only if the release of AIBN from the contact lens lasts for a long enough time to initiate the DMA film. Therefore, a modified method has been proposed by loading vitamin E (α-tocopherol) into contact lenses in order to slow the release of AIBN through the lens network. Specifically, vitamin E acts as an effective diffusion barrier, which forces AIBN through long and tortuous paths, thus, favoring a controlled release of the thermal initiator.

From the literature [38,39,40], it is evident that therapeutic contact lenses based on thin films are still not very widespread. However, it is desirable to investigate their use as ocular drug delivery systems, as they ensure patient comfort due to the flexibility, reduced thickness and non-invasive encumbrance of the films [41]. Moreover, drug release from this kind of platform can be tuned by producing films based on polymeric blends, which also allow to improve the physical and mechanical properties of the films. Easy handling during production, transport and use of film-based systems are also ensured, as well as moderate costs in the formulation development [41].

Recently, polyvinyl alcohol (PVA)/collagen membranes have also been proposed by Daza et al. [42] as a carrier for ciprofloxacin hydrochloride, in order to provide sustained antibacterial activity in the treatment of ulcerative keratitis. Despite the opacity caused by a heterogeneous morphology, the produced membranes were characterized by adequate mechanical strength, water content, hydrophilicity, water vapor permeability and surface pH, guaranteeing the proper comfort. Furthermore, the presence of collagen in the membranes was observed to help reduce stromal damage and improve epithelial regeneration. The results encourage the application of membranes as a cost-effective and safe alternative for the treatment of corneal ulcers.

Table 2 provides an overview of studies focusing on the fabrication of therapeutic contact lenses. The polymeric supports employed to fabricate the contact lenses, the selected active compounds and the technique used for impregnating the drug in the supports, also specifying the final medical application of the ophthalmic drug delivery system.

## 3. Methods of Loading Active Principles into Contact Lenses

Over the years, several approaches have been proposed for impregnating/incorporating ophthalmic drugs into polymeric reservoirs, including commercial contact lenses, hydrogels or films. In Table 3, there is a list of the commercial contact lenses mainly employed as ophthalmic drug delivery platform in the studies, analyzed in this review and previously summarized in Table 2. In addition to the brand name, the manufacturer of the contact lenses and the materials constituting them are also specified [73]. Table 4 reports instead a summary of the different methods employed to develop therapeutic contact lenses, indicating the specific advantages and disadvantages. Each method is detailed in the following subsections.

### 3.1. Soaking Method and Solvent Casting

To date, the soaking method is the common strategy employed to load active compounds mainly into hydrogel-based contact lenses [44,45,46,47,48,49,50], and to a lesser extent, into polymeric films [38,39]. The soaking method consists in immersing the lens/support in a solution/suspension/emulsion containing the drug to be loaded [74]. Absorption of the drug occurs due to the different concentration of the active ingredient in the soaking solution and in the polymer matrix. The study of Xu et al. [20] reports a comparison between the use of a solution or microemulsion to soak contact lenses with an anti-glaucoma drug, namely Bimatoprost. The absorption of the drug using the microemulsion was twice as high as that obtained using the solution, without altering lenses’ properties like swelling, transmittance and folding endurance. More prolonged release kinetics was also achieved by soaking contact lenses using the microemulsion than the other route.

In general, the penetration of the drug into the lens matrix by the soaking method is strongly influenced by the time the contact lens is immersed in the loading solution and the concentration of the drug in the latter. Although the soaking method is very simple and inexpensive, the soaked therapeutic contact lenses are characterized by a great limitation [24,26,33,48,58]: A high initial burst release, associated with a high quantity of ophthalmic drugs impregnated on the external surface of the support and not deep inside the polymeric matrix. In many cases, 90–95% of the ophthalmic drug loaded by soaking method was released from the contact lenses in a very short time, namely in a very few hours [24,26,33,48,58]. This rapid release kinetics is not suitable for the treatment of several chronic diseases, including glaucoma, which is one of the most commonly studied.

Different routes have been attempted to overcome the main drawback associated with the use of the soaking method and, therefore, to prolong the drug release; as first, the incorporation of drug-loaded films, generally produced directly by solvent casting, into the contact lens matrix [27,29,30,31,32]. Until now, drug-loaded films were often incorporated as composite systems into hydrogel contact lenses [27,29,30,31,32]. Although proposed in a limited number of papers [38,39,40], the direct use/wear of polymeric thin and flexible films loaded with ophthalmic drugs as novel therapeutic contact lenses can be very interesting, i.e., assuring comfort and easy handling for the patient consumer.

### 3.2. Loading of Vitamin E into Therapeutic Contact Lenses

A promising approach in prolonging drug release from therapeutic lenses has been found to be vitamin E loading as a diffusion barrier, especially for hydrophilic compounds [35,36,40,50,51,52]. Incorporating vitamin E into contact lenses also brings additional therapeutic benefits, being a powerful antioxidant compound. Several studies highlight the potential of vitamin E to inhibit various ocular diseases, including keratocyte apoptosis as well as the prevention and treatment of cataracts [36,75,76,77,78,79,80,81]. In general, it was observed that by increasing the quantity of loaded vitamin E, the drug release rate was reduced [36,51]. Specifically, in the study of Peng et al. [36], the increase in the release duration of drugs (i.e., fluconazole, dexamethasone, timolol maleate) was found to be quadratic with the increase vitamin E loading, in agreement with the proposed mathematical models. However, the loading of vitamin E has to be optimized considering also a possible deterioration of other properties of the contact lenses, mainly the lens transparency [52] and the oxygen permeability [36]. For example, by loading vitamin E into contact lenses, Peng et al. [36] observed a slight increase in the lens size, a reduction in the oxygen diffusion (about 40%) and in the ion permeability (about 50%), in addition to a beneficial effect of blocking UV radiation that reduce the corneal damage. Similarly, some researchers have also proposed incorporating vitamin A [50] or fatty acids [49] as adjuvant agents to hinder rapid drug release from contact lenses.

### 3.3. Incorporation of Drug-Loaded Nanocomposites or Ring Implants into Contact Lenses

**Figure 4:**
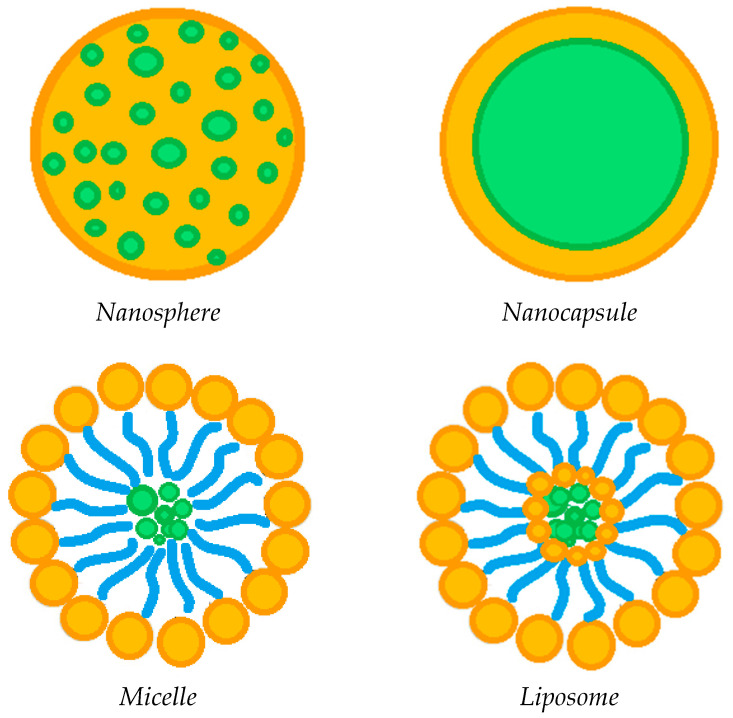
Possible drug-loaded structures incorporated into therapeutic contact lenses.

The incorporation of various drug-loaded structures (Figure 4) into the lens network has been extensively explored as a valid alternative to soaking method, in order to prolong the release of ophthalmic drugs, including:

- drug-loaded nanoparticles [4,19,21,25,37,58];
- drug-loaded liposomes [39,55,56];
- drug-loaded micelles [23,54,82];
- drug-loaded implants [24,25,26,59], generally in the form of rings.

The incorporation of circular or semi-circular ring implants loaded with ophthalmic drugs into contact lenses was proposed in a limited number of papers to extend drug release duration [24,25,26,59]. Nevertheless, this approach seems to be less effective compared to the dispersion of the drug-loaded nanoparticles into the contact lenses network, in order to promote a prolonged drug release [4,19,21,25,37,58]. Specifically, polymer carrier-based nanoparticles can be divided into nanospheres, in which the drug is homogeneously dispersed in a polymeric matrix, or nanocapsules, consisting of a drug core and a polymer shell. The drug-loaded nanoparticles are generally incorporated into the contact lens network by the soaking method [37,57]. The study of Maulvi et al. [19] showed that it is possible to modulate the drug release by changing the polymer/drug ratio used to produce composite nanoparticles. In particular, the dissolution of timolol maleate was prolonged and the burst-like effect reduced by increasing the ethyl cellulose/timolol ratio from 1/1 to 3/1 *w/w*. Drug release was further extended when the 3/1 *w/w* ethyl cellulose/timolol nanoparticle ratio was loaded into ring implants, then incorporated into hydrogel contact lenses. A pH triggered controlled drug release from contact lenses can also be promoted by preparing nanoparticles based on pH-sensitive polymers, such as Eudragits [19]. Loading of precious metal-based nanoparticles has also been attempted in some cases [43,58]. For example, Huang et al. [43] incorporated *N*-[(2-hydroxy- 3-trimethylammonium) propyl] Chitosan Chloride (HTCC) and silver nanoparticles as antimicrobial agents for the treatment of fungal keratitis, in addition to voriconazole (i.e., the drug antifungal model) loaded in graphene oxide. On the other hand, Maulvi et al. [58] proposed the incorporation of gold nanoparticles, together with timolol maleate, in contact lenses. The presence of gold nanoparticles did not affect the swelling and optical transmittance of the contact lenses, while high drug loadings were observed. Unfortunately, there was no significant change in the rate of dissolution of the drug, which was very rapid anyway. Generally speaking, the incorporation of drug-loaded nanoparticles may have a negative influence on some lenses’ properties. In this context, Jung et al. [57] proved that undesired effects including the reduction in ion and oxygen permeability are proportional to the particle loading. Therefore, the loading of nanoparticles has to be optimized not only in terms of drug release duration, but considering, at the same time, the preservation of the fundamental lenses’ features.

Among the various nanometric systems, liposomes already stand out as promising for ocular drug delivery, due to their biocompatibility and ability to increase drug penetration into ocular tissues [83,84,85]. Liposomes are spherical amphipathic vesicles, characterized by a double layer of phospholipids with an internal aqueous cavity. The peculiar structure of liposomes allows the site-specific delivery of both hydrophilic and hydrophobic drugs. Some studies [39,55,56] have shown that the incorporation of drug-loaded liposomes into contact lenses is a promising route to prolong the release of the ophthalmic drug, thus, reducing the administration frequency in the case of chronic ocular pathologies. Specifically, Danion et al. [55] incorporated liposomes containing levofloxacin on the surface of contact lenses by multilayer immobilization. This approach was revealed to be more effective than the soaking method. Indeed, the drug was released from the soaked lenses more or less instantaneously; on the contrary, the presence of liposome layers provided a sustained release of the antibiotic for 6 days. In vivo tests also showed that contact lenses with immobilized liposome layers allowed to control the release of levofloxacin, ensuring topical antibacterial activity over a long period of time. However, at the same time, the liposome loading has to be optimized to ensure crucial properties of the contact lenses, including optical transparency and wettability. An innovative and completely different process for loading liposomes with antibiotics was used by Campardelli at al. [85], which produced liposomes containing ampicillin and ofloxacin using a supercritical CO_2_ based one-step continuous process, named Supercritical Assisted Liposome formation (SuperLip).

Although, to a limited extent, other types of lipids (e.g., triglycerides) have also been employed to prepare solid lipid nanoparticles (SLNs) for the delivery of ocular drugs [37]. The purpose of proposing drug-loaded SLNs is to overcome the drawbacks associated with other colloidal carriers, such as liposomes. Indeed, compared to liposomes, SLNs have numerous advantages, including an easy and economical preparation without the use of organic solvents [84,86,87]. SLNs consist of solid fats (in the range 0.1–30% by weight) dispersed in an aqueous phase.

Another emerging ocular vehicle are micelles [17,54,82,88,89,90,91], which consist of core/shell structures formed by self-assembly. They are generated by the dispersion of amphiphilic molecules; that is, both hydrophobic and hydrophilic compounds in one solution [84,92]. Polymer micelles have high stability and are capable of encapsulating hydrophobic compounds in the core, promoting controlled or targeted release. The presence of surfactants in contact lenses has been shown to be advantageous to control the release of ophthalmic drugs, to increase their corneal permeability, and consequently, their bioavailability, as well as improve their wettability, lubrication and comfort [93,94,95]. Therefore, some studies have attempted to attenuate drug release from contact lenses by incorporating drug-loaded micelles [23,54]. Specifically, Lu et al. encapsulated a hydrophobic fluorescent dye [23], and thus, dexamethasone acetate [54] in the core of the cross-linked micelles, prior to their incorporation into the hydrogel network. This approach promoted a prolonged release of both the dye used as a model compound and the anti-inflammatory drug for at least 14 days, and up to 30 days, respectively. Furthermore, the researchers indicated that the surface wettability and optical transparency of the hydrogels were not adversely affected by the incorporation of drug-loaded micelles [54].

### 3.4. Molecular Imprinting

Molecular imprinting is another novel method recently employed to prepare therapeutic contact lenses [22,60]. It consists of the addition of a template molecule (specifically, the ophthalmic drug) to a monomer solution, with the aim of inducing a spatial arrangement of the monomers according to their ability to interact with the drug-template [96,97,98]. The subsequent steps involve polymerization, cross-linking and finally removal of the template, resulting in the formation of ‘‘cavities” in the polymeric network. In particular, these ‘‘cavities” have adequate dimensions and shapes, which are specific for the drug used as template. Consequently, the imprinted cavities should possess a high affinity for the drug of interest. When imprinted systems are employed for drug delivery, a sustained-release of the drug-template is generally promoted because of the polymer–drug interactions. For example, the advantage of using imprinting technology is evident from the results of the study of Varela-Garcia et al. [60], which focused on the development of hydrogel contact lenses with a strong affinity for acyclovir and its prodrug valacyclovir, generally prescribed to the treatment of herpes simplex virus in the eye. In particular, the valacyclovir loading was significantly higher than the amount of loaded acyclovir, due the stronger interactions of valacyclovir with the methacrylic acid contained in the hydrogel network. Furthermore, acyclovir was completely released from the imprinted contact lenses in just 4 h, while valacyclovir took about 10 h. Therefore, in the case of molecular imprinting, it is clear that drug loading and release control are remarkably influenced by the affinity and formation of interactions between the polymer selected for the lens support and the ophthalmic drug. Moreover, for the preparation of molecular imprinted contact lenses, the template (i.e., the drug) has to be stable under the polymerization conditions and no toxic solvents have to remain on/in the ocular drug delivery system. Commercial contact lenses are structurally made up of several co-monomers and cross-linkers with specific chemical and physical functionalities. Due to the low cross-linking density, the mobility of the polymeric chains that constitute the contact lenses and the free volume between the chains remain [72]. These available and “loose” chains can still reorganize and, even, establish specific interactions with some polymeric regions. These physical rearrangements and reorganization phenomena are called “post-imprinting”.

### 3.5. Supercritical CO_2_—Assisted Technologies

To overcome the main limitations associated with the use of the traditional soaking method and the conventional molecular imprinting, Yañez et al. [72] developed an innovative supercritical fluid−assisted molecular imprinting method. According to the authors, the supercritical procedure allows therapeutic contact lenses to be prepared in shorter process times than those of the conventional molecular imprinting method. In particular, in order to improve flurbiprofen loading and release capability of commercial contact lenses, the supercritical impregnation of the ophthalmic drug and a supercritical fluid extraction step were sequentially performed. In particular, the extraction step was proposed as a drug removal method. Contact lenses processed with the supercritical fluid showed a recognition capacity and a very high affinity for flurbiprofen in aqueous solutions, suggesting the creation of molecularly imprinted cavities, caused by both physical (i.e., swelling/plasticization) and chemical interactions.

Recently, the impregnation using supercritical carbon dioxide (scCO_2_) has been proposed to produce therapeutic contact lenses in different studies [61,62,63,64,65,66,67,68,69,70,71]. Briefly, the scCO_2_ is employed as a solvent to dissolve, and then, to incorporate ophthalmic drugs into the polymer network of contact lenses. The addition of cosolvents, mostly ethanol, was also proposed to enhance the impregnation yields [61,65,66,69]. Almost all papers are focused on the supercritical impregnation of active compounds in hydrogels [61,62,63,64,65,66,67,69]. While, only Duarte et al. [68] have proposed drug-impregnated polymeric films for ocular drug delivery. Some studies have shown that drug loadings generally obtained by supercritical impregnation are remarkably higher than those reached using the soaking method [61,62]. This experimental evidence is attributable to the peculiar characteristics of scCO_2_, for example, the diffusivity, which is comparable to that of gases [99,100]. The drug incorporation into the polymeric network by supercritical CO_2_, and consequently, the drug release from contact lenses can be modulated by changing different process conditions, such as temperature, pressure and depressurization rate [63]. In 2015, Yokozaki et al. [63] demonstrated that increasing the pressure or decreasing the temperature, resulted in an increase in the amount of salicylic acid loaded in the contact lenses. In general, the operating temperature and pressure influence the drug solubility in scCO_2_, which strongly affects the supercritical impregnation of the drug into the polymeric matrix. Another experimental evidence, reported in the paper by Yokozaki et al. [63], is that the quantity of impregnated drug decreased by increasing the depressurization rate, which also induced the collapse of the microstructure of the contact lenses. The study of Masmoudi et al. [64] showed that the supercritical impregnation of cefuroxime sodium, an ophthalmic drug, into contact lenses allow to prolong significantly the drug release, up to several days. However, it was also highlighted that the undesired foaming phenomenon of the polymer can occur in the presence of scCO_2_ under certain conditions. Indeed, it is well-known in the literature that carbon dioxide at supercritical conditions is a foaming agent for some polymers, and thus, modifies their surface structure [101]. In the case of contact lenses, the polymer foaming has to be avoided because it compromises one of the most important functional features of the contact lenses, namely the optical transparency. As also suggested in different papers [64,66,71], the polymer foaming can be avoided by controlling the pressurization and depressurization rates; e.g., by conducting a slow depressurization. Alternatively, the polymers for the preparation of the lens support have to be carefully selected.

### 3.6. Sterilization Step and Post-Processing Stages

Other steps involved in the production and marketing of therapeutic contact lenses have also been further improved, namely the sterilization, packaging and storage of the lenses, to avoid an early and unwanted release of the drug [19,24,25,26,33,59]. Contact lenses are typically sterilized by the wet sterilization process in an autoclave, which involves the drug leaching [24,59]. Galante et al. [33] investigated the influence of the sterilization method on the performance of therapeutic contact lenses prepared by loading different drugs (i.e., levofloxacin, chlorhexidine, diclofenac, timolol) into silicone-based hydrogels. Three different sterilization approaches were investigated, including steam heat, γ-irradiation and ozone gas. Tests on swelling and mechanical properties showed that all sterilization methods led to the formation of drug-polymer interactions, which resulted in a decrease in the amount of drug released by the contact lenses. In addition, steam heat sterilization was shown to lessen the device performance, while irradiation and ozone led to significant degradation of all drugs studied. On the other hand, in the studies by Desai et al. [24,59], the wet sterilization process resulted in greater drug loss than UV radiation sterilization. Similarly, Maulvi et al. [26] has overcome drug leaching associated with the wet process by treating contact lenses in the dry state using radiation sterilization, followed by packaging under aseptic conditions, thus, and avoiding the drug loss that occurs under hydrated conditions [59]. In conclusion, the prevention of drug loss during the sterilization and packaging process can still be considered a challenge.

## 4. Market Outlooks for Therapeutic Contact Lenses

Although being mostly in the preclinical or clinical study stages [12], the main companies competing in the global market of the therapeutic contact lenses are Johnson & Johnson Vision Care Inc. (Jacksonville, FL, USA), UltraVision CLPL (Leighton Buzzard, UK), Unilens Corporation (Clearwater, FL, USA), Bausch & Lomb Incorporated (Rochester, NY, USA), Vistacom Inc. (Allentown, PA, USA), Alcon Pharmaceuticals Ltd. (Fribourg, Switzerland), among others [73,102].

Looking at the current scenario, it is certain that the global market of the drug-loaded contact lenses, also known bandage lenses due to their therapeutic benefits, will experience a strong increase in the coming years. This growing interest is driven by several factors, such as the population aging, the possibility to enhance the re-epithelialization rate of eye tissues, the increased cases of eye disorders/diseases, such as glaucoma or diabetic retinopathy, as well as the increase in the number of surgeries to correct vision or for cataracts, requiring post-operative treatments. In addition to the increase of total health expenditure of all countries for these purposes, the demand for therapeutic contact lenses is also fueled by their ability to reduce the patient discomfort.

The choice of the proper therapeutic contact lenses primarily depends on the pathology of interest. However, in general, there are some fundamental pre-requisites that the therapeutic contact lenses must be strictly adhered to, which also remain current challenges [102]:

- High oxygen permeability.
- Cost-effectiveness.
- The range of some parameters, mainly the back-optic zone radius (BOZR) and total diameter (TD). In general, soft lenses with standard TDs are used; however, in some cases, lenses with larger size may be necessary, for example to prevent wound bleeding after surgery. Consequently, to assure the desired physical fit, the contact lenses with larger TDs required a flatter BOZR.
- Stability of the contact lens on the eye, guaranteed by the minimal dehydration of the hydrogel that usually occurs after the lens application. However, this aspect is a serious problem, for example, for patients suffering from the dry eye syndrome, among other disorders.
- To minimize the deposition of impurities on the lens surface, which should ideally be resistant to its formation. A practical route could be the use of disposable lenses, but the patient compliance and the efficacy of therapeutic treatment could be reduced.

Some regulatory constraints about the marketing of therapeutic contact lenses have also to be taken into account [12]. A primary regulatory aspect is to understand if the therapeutic contact lenses have to be considered a drug or a combination product. If the lens is considered only a support for the ophthalmic drug delivery, the product would be likely considered a drug from a regulatory point of view. On the other hand, if the contact lenses are also a device with its own functions (for example, refraction correction), it would be more properly considered as a combination product. Moreover, a common approach is to load drug molecules already approved by US Food and Drug Administration and to develop a novel ophthalmic drug delivery platform. However, additional preclinical and clinical studies would be required regarding the safety, efficacy, and pharmacokinetics also in the case of the novel product.

## 5. Conclusions

The use of contact lenses as a platform for ocular drug delivery is an innovative and effective strategy for treating different ocular pathologies, and overcoming the drawbacks associated with the administration of common eye drops. However, more research needs to be conducted for marketing of drug-loaded contact lenses to ensure efficacy, safety and comfort for consumers. The different studies analyzed in this review clearly highlight that the main challenge is still to promote a prolonged release of ophthalmic drugs from contact lenses. Indeed, the soaking method, as an easy and common route to incorporate active compounds into contact lenses, results in low drug absorption and high burst release. Among the alternative approaches, the supercritical impregnation of drugs into contact lenses and the incorporation of vitamin E as a barrier to diffusion and as an adjuvant active compound for the treatment of ocular diseases have proved very promising in prolonging drug release. To this end, it has been found that the incorporation of drug-loaded liposomes and micelles into contact lenses is also a good approach to avoid drug leaching that characterizes soaked lenses, but further investigations need to be conducted given the limited number of studies available. The recently proposed molecular imprinting using scCO_2_ is also interesting in overcoming some limitations of the conventional molecular imprinting, but further studies focused on its application are needed. It is in fact essential to point out that, in addition to ensuring high drug loadings and sustained release, a good method for the fabrication of therapeutic contact lenses has also to guarantee fundamental properties, such as the transparency of the lens. Generally speaking, the drug incorporation into hydrogel-based contact lenses has been widely employed; conversely, the use of thin polymeric films as supports for therapeutic lenses needs to be further investigated. Indeed, thin films can increase the patient comfort and, when based on polymeric blends, can efficiently tune the drug release. Other stages, such as sterilization, packaging and storage of therapeutic contact lenses have also been further improved, to avoid the premature and undesired release of the incorporated drug.

## Figures and Tables

**Table 1:** A list of the polymers mainly employed to prepare therapeutic Contact Lenses (CLs). pHEMA: poly (2-hydroxyethyl methacrylate); PMMA: poly (methyl methacrylate).

Polymer	Properties
pHEMA	Biocompatible, but not biodegradable Hydrophilic properties, due to the presence of an –OH group Water-absorbing material When dry, it has the properties of hard organic glass; after the hydration, it becomes soft and flexible Generally display very poor mechanical properties Generally employed for soft CLs, which are fragile/less durable but very comfortable
PMMA	Rigid/poorly flexible High stability to UV and atmospheric agents Limited chemical and heat resistance Scarce permeability to oxygen Excellent light transmission Good mechanical and optical properties (e.g., transparency) Low water-absorbing capacity Generally used for rigid CLs, which are cheap and durable, but uncomfortable
Silicone/siloxane	Biocompatible, but not biodegradable Chemical inert (e.g., not readily attracked by oxygen) Resistance to water and oxidation Highly flexible Stability at both high and low temperatures Very high permeability to gases Generally employed for soft CLs, which are fragile/less durable but very comfortable

**Table 2:** Preparation of therapeutic contact lenses (CLs). AIBN: azobisisobutyronitrile; APMA: aminopropyl methacrylamide; β-CD: β-cyclodextrin; BEM: 2-butoxyethyl methacrylate; BSA: bovine serum albumin; DDAO: 7-Hydroxy- 9H-(1,3-dichloro-9,9- dimethylacridin-2-one); CMC: carboxymethyl chitosan; DEAA: *N,N*-diethylacrylamide; DMA: dimethylacryamide; DMPC: dimyristoyl phosphatidylcholine; EC: ethyl cellulose; EGDMA: ethylene glycol dimethacrylate; GMA: glycidyl methacrylate; GO: grapheme oxide; HEMA: poly (hydroxyethyl methacrylate); HPMC: hydroxypropyl methylcellulose; HTCC: *N*-[(2-hydroxy- 3-trimethylammonium) propyl] Chitosan Chloride; MAA: methacrylic acid; MMA: methylmethacrylate; NCs: nanocrystals; NPs: nanoparticles NVP: *N*-vinyl pyrrolidone; PEG-b-PCL: polyethylene glycol-block-polycaprolactone; PCL: polycaprolactone; PGT: propoxylated glyceryl triacrylate; PLGA: poly(lactide- co-glycolide); PMMA: poly (methyl methacrylate); P(MMAEHA-EGDMA): poly (methylmethacrylate-coethylhexylacrylate-co-ethyleneglycoldimethacrylate); PVA: polyvinyl alcohol; rAAV: recombinant adeno-associated virus; SiMA: 1-(tristrimethyl-siloxysilylpropyl)- methacrylate.

Drug-Loading Method	Polymeric Support	Active Principle	Application	Outcome	Ref.
Soaking method	Polyurethane film produced by solvent casting	Brimonidine tartrate	Glaucoma	Prolonged drug release up to 14 days	[38]
Drug-loaded liposomes by hydration method; soaking method	Lipids-based film	Besifloxacin hydrochloride	Conjunctivitis	Biphasic release: initial burst + sustained (80% released in 10 h)	[39]
-	DMA film placed on commercial CLs loaded with AIBN	Loading of vitamin E	-	AIBN release in about 30 min	[40]
Solvent casting	PLGA film embedded in methafilcon hydrogel- based CLs	Dexamethasone	Retinal diseases, such as diabetic macular edema	Prolonged drug release up to 160 h	[30]
Solvent casting	PLGA film embedded in HEMA hydrogel-based CLs	Ciprofloxacin	Post-operative treatment	Prolonged drug release for 1 month	[27]
Solvent casting	PLGA film embedded in HEMA hydrogel-based CLs	Econazole	Fungal keratitis	Extended antifungal activity	[29]
Solvent casting	PLGA film embedded in methafilcon hydrogel- based CLs	Latanoprost	Glaucoma	Initial burst + sustained drug release for 1 month.	[31]
Solvent casting	PLGA film embedded in methafilcon hydrogel- based CLs	Latanoprost	Ocular hypertension, glaucoma	Sustained drug delivery as effective as eyedrops	[32]
Solvent casting	Hydrogel-based CLs	Voriconazole loaded into GO; HTCC and silver nanoparticles as antimicrobial agents	Fungal Keratitis	Good antifungal and antimicrobial activity	[43]
Soaking or mixing method	PVA/collagen membrane	Ciprofloxacin hydrochloride, tobramycin	Ulcerative keratitis	Antibacterial activity for 48 h	[42]
Soaking method	HEMA hydrogels with/without APMA	rAAV	Corneal gene therapy	Efficacy in transduction/ triggering cell proliferation	[44]
Soaking method	Silicone hydrogels	Levofloxacin, chlorhexidine, diclofenac, timolol	-	High burst in the release profiles, optimization of sterilization method	[33]
Soaking method	Hydrogels based on HEMA, silicone, NVP and MMA	HPMC	-	pH-sensitivite drug release	[28]
Soaking method	PEG-modified silicone hydrogel	Roscovitine	Retinoblastoma	Prolonged drug release	[34]
Soaking method	HEMA/β-CD- hyaluronan hydrogels	Diclofenac sodium	Conjunctivitis	Therapeutic effect for conjunctivitis	[45]
Soaking method	Commercial hydrogel- based CLs	Voriconazole, diclofenac sodium	Acanthamoeba keratitis	Sustained release, cell proliferation	[46]
Soaking method	HEMA hydrogel- based CLs	Triamcinolone acetonide	Allergy	Prolonged drug release	[47]
Soaking method	Commercial HEMA hydrogel- based CLs	Timolol maleate	-	High-burst: 95% of drug released in 2 h	[48]
Soaking method from a solution or microemulsion	CLs based on siloxane, DMA, EGDMA, HEMA, Irgacure	Bimatoprost	Glaucoma	Microemulsion better than solution to prolong release	[20]
Soaking method	Commercial CLs based on silicone or HEMA hydrogels	Tetracaine, bupivacaine, ketotifen, diclofenac, flurbiprofen; loading of fatty acids (i.e., oleic acid, linoleic, α-linolenic acid)	-	Prolonged drug release, with an initial burst in the range 30–90% depending on the drug/CLs system	[49]
Soaking method	Commercial silicone hydrogel CLs	Timolol maleate; loading of vitamin E	Ocular hypertension, glaucoma	High drug bioavailability, reduction of hypertension	[35]
Soaking method	HEMA-based hydrogels with EGDMA, GMA, NVP, AIBN	Alexa Fluor 488 dye, brimonidine, timolol; loading of vitamin E and A	Glaucoma	Increase in drug loading, drug release in 6 h	[50]
Soaking method	Commercial silicone hydrogel CLs	Fluconazole, dexamethasone, timolol maleate; vitamin E loading	Eye inflammation, glaucoma	Prolonged drug release, beneficial effect of blocking UV radiation	[36]
Soaking method	Commercial CLs based on senofilcon A	Timolol maleate, dorzolamide hydrochloride; vitamin E loading	Glaucoma	Prolonged drug release	[51]
Soaking method	Commercial silicone hydrogel CLs	Dexamethasone; vitamin E loading	Eye inflammation	Prolonged drug release	[52]
Drug/BSA NCs by acid-base neutralization reactions; soaking method	NCs based on BSA and loaded into HEMA hydrogels	Meloxicam	Ocular irritation, antophthalmia after cataract surgery	Reduction of eye irritation, extended drug release	[53]
Drug-loaded micelles by thin-film hydration method	PEG-b-PCL micelles (with a PCL core and silica shell) loaded into HEMA hydrogels	Dexamethasone	Chronic posterior segment eye diseases	Prolonged drug release up to 30 days	[54]
-	PEG-b-PCL micelles into HEMA hydrogels	DDAO dye	-	Extended release for at least 14 days	[23]
Drug-loaded liposomes placed on CLs by multilayer immobilization; soaking method	Commercial CLs	Levofloxacin	Bacterial infections, such as keratitis	Antibacterial activity against Staphylococcus aureus	[55]
Drug-laden liposomes loaded into hydrogels by free radical solution polymerization	DMPC liposomes loaded into HEMA-hydrogel CLs	Lidocaine	-	Drug release prolonged for about 7 days	[56]
Drug-loaded lipid NPs by melt-emulsification and ultrasonication method; soaking	Compritol 888 ATO and triglyceride as lipid carriers for NPs soaked into hydrogels based on CMC and poloxamer 407	Quercetin	-	Improvement in drug transcorneal penetration and the precorneal retention time	[37]
Drug/PGT NPs by thermal polymerization; soaking method	PGT-based NPs loaded into commercial silicone hydrogel CLs	Timolol maleate	Ocular hypertension, glaucoma	Prolonged drug release, reduction in hypertension, optimization of storage conditions	[57]
Drug/silica NPs by microemulsion	Drug/silica shell NPs loaded into HEMA-based hydrogels	Lidocaine	Glaucoma	Prolonged drug release kinetics, with a 50% burst effect	[4]
Drug/Eudragit NPs by quasi-emulsion solvent diffusion; soaking for direct- drug-loaded CLs	pH-sensitive drug/Eudragit S100 NPs laden into hydrogel CLs	Cyclosporine	Chronic dry eyes syndrome	Prolonged drug release up to 14 h, no leaching after packaging	[19]
Drug/silica NPs by microemulsion; direct-drug-loaded CLs by free radical polymerization	Drug/silica shell NPs loaded into hydrogels based on HEMA, MAA, EGDMA	Ketotifen fumarate	Allergy	Reduced loss in transmittance and physical properties of hydrogels with NPs by emultion	[21]
Drug-loaded EC NPs by double emulsion	Drug/EC NPs into ring implants based on HEMA, EGDMA and MAA, then incorporated into hydrogel CLs	Timolol maleate	Glaucoma	Sustained drug release and reduction in intraocular pressure (for 170 h /190 h)	[25]
Soaking method	CLs based on HEMA, DMA, EGDMA, NVP, siloxane and Irgacure	Timolol maleate, gold NPs	-	Rapid drug release in a few hours, reduction in intraocular pressure	[58]
Soaking method	Drug-loaded semi-circular rings based on HEMA, MAA, EGDMA and Irgacure D, then implanted into hydrogel CLs	Moxifloxacin HCl, hyaluronic acid	Conjunctivitis	Improvement in drug residence time, bactericidal activity, optimization of sterilization method	[26]
Cast moulding	Drug- loaded semi-circular acrylate rings implanted in CLs	Timolol maleate, hyaluronic acid	Glaucoma	High burst in drug release, optimization of sterilization	[59]
Solvent casting for drug-loaded implants; soaking method for direct-drug-loaded CLs	Silicone CLs; implants based on Irgacure, EGDMA, DMA, NVP, siloxane, HEMA, then embedded into silicone CLs	Bimatoprost, hyaluronic acid, timolol	Ocular hypertension, glaucoma	High burst effect in drug release profiles	[24]
Molecular imprinting	Hydrogels based on HEMA, DEAA, DMA, SiMA, MMA	Timolol maleate	Glaucoma	Fast release, increase in drug loading by imprinting	[22]
Molecular imprinting	Hydrogels based on HEMA, EGDMA, MAA, AIBN	Acyclovir, valacyclovir hydrochloride	Herpes simplex virus ocular keratitis	High drug loadings	[60]
Soaking method; supercritical impregnation	Commercial hydrogel CLs based on Methafilcon A, Nelfilcon A, Omafilcon A, Hilafilcon B	Flurbiprofene, timolol maleate	Glaucoma	Higher drug loadings with shorter process times by sup. impregnation	[61]
Soaking method; supercritical impregnation	Foldable acrylic hydrogel CLs, based on HEMA and BEM with MAA and acrylamide	Norfloxacin	Cataract	Higher drug loadings by supercritical impregnation	[62]
Supercritical impregnation	Commercial silicone CLs based on Hilafilcon B	Salicylic acid	-	Prolonged drug release for 8 h	[63]
Supercritical impregnation	Commercial rigid CLs based on PMMA	Cefuroxime sodium	Cataract	Prolonged drug release for 15 days	[64]
Supercritical impregnation	Commercial silicone hydrogel CLs, based on Balafilcon A	Acetazolamide, timolol maleate	Glaucoma	Higher drug loadings	[65]
Supercritical impregnation	Foldable acrylic CLs, based on HEMA	Dexamethasone, ciprofloxacin	Cataract	Prolonged drug release for 60 days	[66]
Supercritical impregnation	Commercial foldable acrylic CLs	Gatifloxacin	Endophthalmitis after cataract surgery	Imrpovement in impregnation yield	[67]
Supercritical impregnation	P(MMA-EHA-EGDMA) films	Flurbiprofen	Eye surgery	Prolonged drug released in 3 months	[68]
Supercritical impregnation	Commercial foldable acrylic CLs	Methotrexate	Posterior capsule opacification after cataract surgery	Prolonged drug release for more than 100 days, inhibition of fibrosis	[69]
Supercritical impregnation	Commercial silicone CLs, based on Balafilcon A	Acetazolamide	Glaucoma	Fast drug release in about 3 h	[70]
Supercritical impregnation	Commercial rigid CLs based on PMMA	Dexamethasone, ciprofloxacin	Prevention of short- and mid-term postoperative complications	Prolonged drug release up to about 40 days	[71]
Supercritical CO_2_- assisted molecular imprinting	Commercial silicone CLs	Flurbiprofen	-	Prolonged drug release up to about 8 h	[72]

**Table 3:** Commercial contact lenses proposed as platform for ocular drug delivery.

Brand	Material	Company
PureVision	Balafilcon A	Bausch & Lomb
Bioinfinity	Comfilcon A	Cooper Vision
Dailies Total	Delefilcon A	Alcon
ACUVUE Advance	Galyfilcon A	Johnson & Johnson
Night & Day	Lotrafilcon A	CIBA Vision
Air Optix Aqua	Lotrafilcon B	CIBA Vision
ACUVUE Tru	Eye Narafilcon A	Johnson & Johnson
ACUVUE Oasys	Senofilcon A	Johnson & Johnson
Clariti 1-day	Somofilcon A	Cooper Vision

**Table 4:** Advantages and disadvantages of the main methodologies used to prepare drug-loaded contact lenses (CLs).

Method	PROS	CONS
Soaking method	Easy, fast and low-cost method to load drugs into CLs	Massive use of solvents Low drug loadings, mainly due to a scarce penetration in the polymeric bulk High burst-effect in the release kinetics Rapid drug release
Solvent casting	Prolonged drug release Possible comfort and easy handling when thin and flexible films loaded with drugs are directly used as CLs	Possible degradation of active compounds due to the high process temperatures
Loading of vitamin E	Prolonged drug release Additional therapeutic properties, mainly antioxidant activity Blocking of UV radiation, which damage eye tissues	Possible worsening of the lens’ properties, as optical transparency, wettability, oxygen permeability A diffusion barrier mainly limited to hydrophilic compound
Incorporation of drug-loaded nanostructures or ring implants	Prolonged drug release	Possible worsening of the lens’ properties, as optical transparency, wettability, oxygen permeability Soaking method (with the related drawbacks) is often involved to incorporate drug-loaded particles
Molecular imprinting	Formation of cavities into the CLs support with proper size/shape and high-affinity for a specific drug High drug loadings Prolonged drug release	Possible undesired post-imprinting phenomena, like rearrangements of polymeric chains The selected drug as to be stable under the polymerization conditions
Supercritical technologies	High drug loadings Prolonged drug release Preservation of polymeric structure	Possible worsening of the lens’ optical properties, mainly due to a possible polymer foaming High operating costs due to high pressures

## Data Availability

Not applicable.
